# Multi-dimensional variables and feature parameter selection for aboveground biomass estimation of potato based on UAV multispectral imagery

**DOI:** 10.3389/fpls.2022.948249

**Published:** 2022-07-29

**Authors:** Shanjun Luo, Xueqin Jiang, Yingbin He, Jianping Li, Weihua Jiao, Shengli Zhang, Fei Xu, Zhongcai Han, Jing Sun, Jinpeng Yang, Xiangyi Wang, Xintian Ma, Zeru Lin

**Affiliations:** ^1^Institute of Agricultural Resources and Regional Planning, Chinese Academy of Agricultural Sciences, Beijing, China; ^2^Collaborative Innovation Center on Forecast and Evaluation of Meteorological Disasters, Nanjing University of Information Science & Technology, Nanjing, China; ^3^School of Remote Sensing and Information Engineering, Wuhan University, Wuhan, China; ^4^Center for Agricultural and Rural Economic Research, Shandong University of Finance and Economics, Jinan, China; ^5^Potato Science Institute, Jilin Academy of Vegetables and Flower Sciences, Changchun, China; ^6^School of Economics and Management, Tiangong University, Tianjin, China

**Keywords:** remote sensing phenotypes, spectral indices, texture, geometric parameters, frequency-domain indicators, variables preference

## Abstract

Aboveground biomass (AGB) is an essential assessment of plant development and guiding agricultural production management in the field. Therefore, efficient and accurate access to crop AGB information can provide a timely and precise yield estimation, which is strong evidence for securing food supply and trade. In this study, the spectral, texture, geometric, and frequency-domain variables were extracted through multispectral imagery of drones, and each variable importance for different dimensional parameter combinations was computed by three feature parameter selection methods. The selected variables from the different combinations were used to perform potato AGB estimation. The results showed that compared with no feature parameter selection, the accuracy and robustness of the AGB prediction models were significantly improved after parameter selection. The random forest based on out-of-bag (RF-OOB) method was proved to be the most effective feature selection method, and in combination with RF regression, the coefficient of determination (R^2^) of the AGB validation model could reach 0.90, with root mean square error (RMSE), mean absolute error (MAE), and normalized RMSE (nRMSE) of 71.68 g/m^2^, 51.27 g/m^2^, and 11.56%, respectively. Meanwhile, the regression models of the RF-OOB method provided a good solution to the problem that high AGB values were underestimated with the variables of four dimensions. Moreover, the precision of AGB estimates was improved as the dimensionality of parameters increased. This present work can contribute to a rapid, efficient, and non-destructive means of obtaining AGB information for crops as well as provide technical support for high-throughput plant phenotypes screening.

## Introduction

One of the 4th largest staples in the world, the potato enjoys an unparalleled position when it comes to food safety (Li et al., 2018b). Aboveground biomass (AGB) is a key metric to evaluate crop performance and is inextricably linked to yield, and its dynamics directly reflect the strength and trophic state of the crop (Zheng et al., 2019). Therefore, accurate and efficient monitoring of AGB can provide timely messages on crop growth and production estimation, which matters to guide fine farming management.

Currently, unmanned aerial vehicle (UAV) remote sensing technology has gained widespread attention in crop AGB monitoring due to the virtues of its flexible application, simple operation, and access to high space–time resolution images (Watanabe et al., 2017; Yang et al., 2017). The multispectral sensors can be compatible with the advantages of the hyperspectral and RGB sensors, such as being economically suitable, containing the red-edge and near-infrared bands, and allowing comparable spectral data to be obtained through radiometric calibration, thus gaining widespread interest in quantitative remote sensing in agriculture (Deng et al., 2018). Therefore, it is necessary to discuss the application of multispectral imagery in AGB estimation (Han et al., 2019).

The parameters that can be extracted from UAV images to characterize crop growth can be broadly classified into the following four categories. (i) Spectral variable (SV): Spectral indices (e.g., vegetation indices, VIs) are the most extensively employed parameters in precision agriculture since they have explicit physical meaning, but for many crops, the accuracy of the model is prone to saturation due to canopy closure during the late growth stage (Zheng et al., 2019). (ii) Texture variable (TV): Textures reflect the gray-scale properties of images and the spatial position of image pixels, which makes it possible to combine them with spectral variables to reduce the underestimation of crop parameters using VIs alone and thus improve the applicability of the estimation model (Li et al., 2020). The most prevalent and effective texture available is the gray level co-occurrence matrix (GLCM). (iii) Geometric variable (GV): Canopy height and fractional vegetation cover (FVC) are frequently used and valid indicators of geometric variables, reflecting the growth of the crop in both vertical and horizontal directions (Wan et al., 2020). (iv) Frequency-domain variable (FDV): The frequency-domain variable is characterized by a spectrum representing the distribution of energy. The algorithm represented by the Fourier transform converts the imagery from space to frequency dimension containing only different frequency information (high- and low-frequency information), which can highlight or suppress the details and noise of the image (Yang et al., 2019).

The joint employment of some of the above variables is presently common in precision agriculture, but few reports reveal the contribution of different dimensional variables and how they were selected. Therefore, with such a large number of variables, it is necessary to effectively extract the most appropriate variables for AGB prediction. The selection of feature variables has rarely been considered in most studies (Zheng et al., 2019; Liao et al., 2020; Maimaitijiang et al., 2020; Wan et al., 2020). The commonly applied methods for feature parameter selection are RReliefF (Li et al., 2020; Acikgoz, 2022) and machine learning (Janitza et al., 2018) such as random forest (RF). However, the difference and effectiveness of these methods for variable selection have been less studied. Moreover, there are few studies on biomass estimation in potato crops and the predictive variables are mainly focused on spectral indices and height (Li et al., 2020).

Considering that few studies have used variables from the above four dimensions simultaneously to predict AGB and to explore the impact of different feature parameter selection methods, in this study, parameters of the spectral, texture, geometric, and frequency domain were extracted from UAV multispectral images and three methods were chosen to calculate the importance of the variables, and finally, the most important parameters were selected to predict potato AGB. The major targets of the article are to (1) extract as many multi-dimensional parameters as possible that have the predictive potential for potato AGB; (2) compare the differences of three feature parameter selection methods in determining the importance of different dimensional variables and their impact on potato AGB estimation; and (3) predict potato AGB with combinations of different dimensional variables and compare their performance.

## Materials and methods

### Experimental design

**Figure 1:**
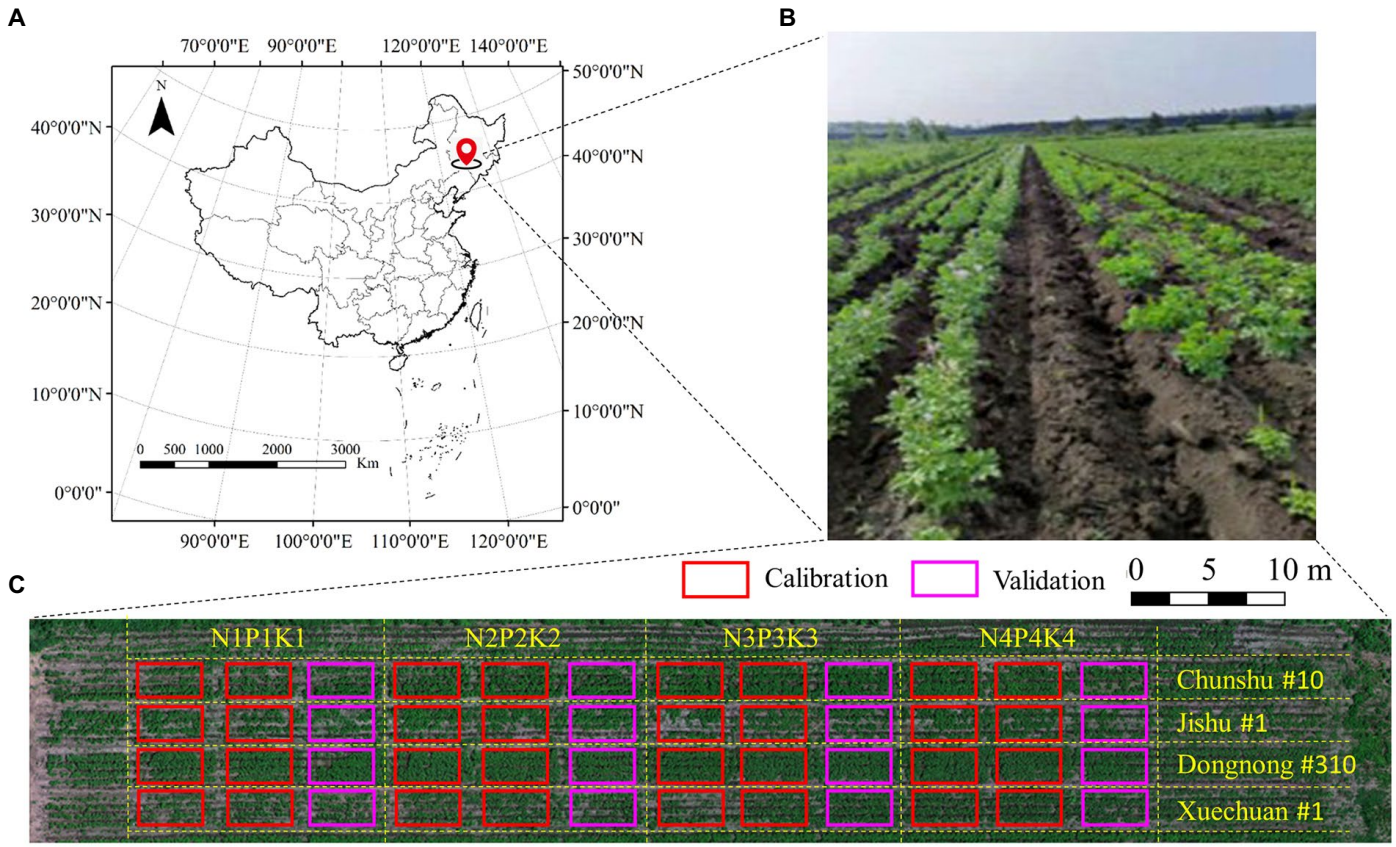
Potato trial layout: **(A)** the trail location; **(B)** the field scene photo; **(C)** experimental design details.

The potato plant trials were conducted from May to August 2021 in Changchun City, Jilin Province, China (43.45°N, 124.99°E). Four widely cultivated varieties (Dongnong #310, Jishu #1, Chunshu #10, and Xuechuan #1) were involved. Different fertilizer treatments (N, P, and K) were used to simulate differentiated field cropping conditions. A total of four gradients (N1P1K1: no fertilization; N2P2K2: half of the normal fertilization; N3P3K3: normal fertilization; and N4P4K4: twice of normal fertilization) and three repetitions were set. The whole experimental area was divided into 48 small plots of the same size, with an area of about 15 m^2^ (6 m × 2.5 m). Figure 1C shows the experimental design details.

### UAV data acquisition

The MS600 PRO multispectral sensor was installed on a DJI Matrice 200 drone at a 40-m altitude to collect the centimeter-level images with an 18.8 mm spatial resolution. Six independent camera lenses [central bands of 450@35 (B), 555@25 (G), 660@20 (R), 720@10 (RE1), 750@15 (RE2), and 840@35 nm (NIR)] were equipped. The camera can realize automatic recognition of gray plate, real-time calculation of reflectance data, and synchronous preservation of reflectance images. In addition, its high-precision radiometric calibration and downline light sensor can ensure that users get a stable and accurate reflectance of ground objects, thus improving the consistency of data acquisition at different times and under different environments. After the route flights (overlap both across-and along-track was 80%, flight speed was 2 m/s), Yusense Map software (Changguang Yusense Information Technology and Equipment Co., Ltd., Qingdao, China) was used to complete data preprocessing and generate DSM data. The process of acquiring reflectance images includes taking vertical downward shots of the matching calibration panels with the UAV in hand before takeoff, importing the original images and the calibration panels images into the software and framing the calibration area, and automatically conducting radiometric calibration and calculating reflectance according to the calibration panel DN values by the software. During the potato growth periods, we completed three flights from 11:00–13:00 on June 18 (seedling period, SP), July 17 (flowering period, FP), and August 9 (tuber period, TP). After obtaining the reflectance images, a rectangular region of interest (ROI) was defined for each plot, and the mean reflectance within the region was treated as the plot-level reflectance of the plot.

### Field data measurement

Field measured data include canopy height, hyperspectral curves of different endmembers, and AGB of each plot. The millimeter-scale ruler was used to measure the true value of potato canopy height at each period. After the execution of each flight, three potato plants in each plot were randomly dug out, then the roots were subtracted, and the rest were dried indoors. These plants were ovened at 110°C for a few minutes before being kept at 75°C until the weight remained unchanged. Finally, the electronic balance was applied to weigh them and AGB was calculated in combination with the planting density. Hyperspectral curves of different endmembers at three stages were measured by the ASD spec four spectrometers.

### Multi-dimensional parameters extraction based on UAV images

After each plot of the imagery was defined to acquire plot-level reflectance, several VIs (plot-level VIs) commonly used in precision agriculture (shown in Table 1) were computed according to the plot-level reflectance.

**Table 1:** VIs of different band combinations for predicting potato AGB.

Vegetation indices	Formula	References
NDVI	(R_840nm_ − R_660nm_)/(R_840nm_ + R_660nm_)	Rouse et al., 1974
NDRE	(R_840nm_ − R_720nm_)/(R_840nm_ + R_720nm_)	Gitelson and Merzlyak, 1997
MTCI	(R_840_ − R_720nm_)/(R_720_ + R_660nm_)	Dash and Curran, 2004
EVI2	2.5(R_840nm_ − R_660nm_)/(R_840nm_ + 2.4_R660nm_ + 1)	Jiang et al., 2008
VARI	(R_555nm_ − R_660nm_)/(R_555nm_ + R_660nm_)	Gitelson et al., 2002
OSAVI	(1 + 0.16)(R_840nm_ − R_660nm_)/(R_840nm_ + R_660nm_ + 0.16)	Rondeaux et al., 1996

In practice, most of the pixels obtained by sensors are mixed pixels, and there is little detailed description information about the components, so it is difficult to give a more accurate description inside the pixels. The linear model is extensively used in spectral mixture analysis (SMA) due to its simpleness and clear physical meanings (Chang, 2017). In this study, the linear model of fully constrained least-square (LM-FCL) was used to obtain pure vegetation information.

(1) $$R_{mp}=\overset{M}{\underset{i=1}{\sum }}Abd_{i}R_{i}+\varepsilon$$

(2) $$0\leq Abd_{i}\leq 1\;$$

(3) $$\overset{M}{\underset{i=1}{\sum }}Abd_{i}=1$$

where *R*_mp_ and *R*_i_ represent the reflectance of the mixed pixel and pure endmember, respectively. Abd is the abundance of different endmembers, M denotes the endmember amounts, and ε shows the error.

GLCM was considered to be the combined likelihood distributed of the pixel couple (Haralick et al., 1973). Six GLCM-based textures, variance (VAR), homogeneity (HOM), contrast (CON), dissimilarity (DIS), entropy (ENT), and second moment (SEC), were selected to participate in the AGB prediction.

Geometric variables, such as canopy height (Jiang et al., 2019) and FVC (Wan et al., 2020), were considered as the important predictors of crop biomass and yield. The canopy height can be accessed by subtracting the ground DEM from DSM. For assurance of the FVC precision achieved, the dimidiate pixel model (DPM) and the image classification method were used to check each other. The blue, green, and red bands were extracted from the multispectral images, and the true color synthesis was realized by RGB superposition. The support vector machine was applied for image classification to extract vegetation parts, and then, the FVC data can be obtained *via* the division of the plant pixel count by the overall. Moreover, the NDVI-based DPM was employed in the FVC estimation (Yan et al., 2022). Equation (4) shows the calculation principle.

(4) FVC = (NDVI_M_ − NDVI_NS_)/(NDVI_PP_ − NDVI_NS_)

where NDVI_M_, NDVI_NS_, and NDVI_PP_ denote NDVI values of mixed, naked soil, and pure plant pixels, respectively. In this paper, due to the inevitable noise, the maximum and minimum values of NDVI_veg_ and NDVI_soil_ were set within the range of 98% confidence.

By transforming each spectral curve into ensembles of sine and cosine functions (see Eq. 5), the spectral domain data [R_j_ = (r_1_, r_2_, …, r_n_), j is the band serial number (j = 1, 2, …, n), r is the reflectance, and n is band number] is converted into the frequency domain, thus obtaining parameters such as constant terms (A_0_/2), amplitude (A_t_, B_t_, C_t_), and phase (φ_t_) that characterize the function (Jiang et al., 2021).

(5) $$\begin{array}{c} r\left(j\right)=\frac{A_{0}}{2}+\overset{\infty }{\underset{t=1}{\sum }}\left[A_{t}\cos\left(2\pi tj/n\right)+B_{t}\sin\left(2\pi tj/n\right)\right]\;\\ =\frac{A_{0}}{2}+\overset{\infty }{\underset{t=1}{\sum }}C_{t}\sin\left(2\pi tj/n+\varphi_{t}\right) \end{array}$$

(6) $$\frac{A_{0}}{2}=\frac{1}{n}\left(r_{1}+r_{2}+..+r_{n}\right)$$

(7) $$A_{t}=\frac{2}{n}\overset{n}{\underset{j=1}{\sum }}r_{j}\cos\left(2t\pi j/n\right)$$

(8) $$B_{t}=\frac{2}{n}\overset{n}{\underset{j=1}{\sum }}r_{j}\sin\left(2t\pi j/n\right)$$

(9) $$C_{t}=\sqrt{\left(A_{t}{}^{2}+B_{t}{}^{2}\right)}$$

(10) $$\varphi_{t}=\arctan\left(A_{t}/B_{t}\right)$$

where *t* is the decomposition times.

### Feature parameter selection methods

#### RReliefF algorithm

RReliefF algorithm is a feature selection method based on statistical correlation (Robnik-Sikonja and Kononenko, 2003). By randomly selecting a sample R in the training set, and then, searching its adjacent samples (the same class H and diverse class M), weights of each feature are updated according to the distance between R, H, and M. For continuous feature values, the difference [*Dif*(F, R_1_, R_2_)] between two samples R_1_ and R_2_ for feature F is defined as:

(11) $$Dif\left(F,R_{1},R_{2}\right)=\frac{\left|R_{1}\left(F\right)-R_{2}\left(F\right)\right|}{\max-\min}$$

where Max and Min represent the maximum value of F.

The weight [W(F)] of feature F can be given by approximate probability distribution:

(12) W(F) = P(*Dif*(F, R_1_, R_2_)|M) − P(*Dif*(F, R_1_, R_2_)|H)

For the regression problem, two resampling probabilities are introduced to judge if they are in the same class. Probabilistic determinations make it possible to model and forecast the corresponding intervals between two resamples.

(13) P_1_ = P(*D*if(F)|H)

(14) P_2_ = P(*D*if(prediction)|H)

where P_1_ and P_2_ are the simulated and predicted values of the distance probability of two similar samples.

According to the conditional probability:

(15) P_2|1_ = P(*D*if(prediction)|*D*if (F)|H)

Combined with Eq. (12) and Eq. (15):

(16) W(F) = (P_2|1_ × P_1_)/P_2_ − [(1 − P_2|1_) × P_1_]/(1 − P_2_)

#### RF algorithm based on Gini index and error of out-of-bag

Bootstrap resampling technology is used in RF to collect a certain amount of samples in the target dataset. In each round of random sampling of bagging, some data in the training set are not selected (out-of-bag, OOB). This part is not engaged in the data simulation and thus serves to check the model’s robustness.

The Gini index selection standard can be expressed that each sub-node reaches the highest purity (Boulesteix et al., 2012), that is, all observations falling in the sub-node belong to the same classification. For the decision tree (DT) in RF, there are v (v = 1, 2, …, q) classes of samples altogether. Assuming a sample falls into class v with probability p_v_, the probability distribution of Gini index [G(PD)] can be defined as Eq. (17).

(17) $$G\left(PD\right)=\overset{q}{\underset{v=1}{\sum }}p_{v}\left(1-p_{v}\right)=1-\overset{q}{\underset{v=1}{\sum }}p_{v}^{2}$$

Procedure for measuring the importance of features through the error of OOB includes:

(i) The OOB data that correspond to every DT was chosen to compute the error (E_1_)
(ii) The noise is appended to F to compute the OOB error (E_2_)
(iii) The assumption is that there are a total of K DTs, the importance of F (IMP_F_) can be calculated:

(18) IMP_F_ = ∑ (E_2_ − E_1_)/K

IMP_F_ is able to account for the importance of F in that if there is a marked reduction in the precision of the OOB data after the addition of noise (i.e., an increase in E_2_), this indicates that F strongly influences the predicted outcome.

### Regression algorithms and accuracy evaluation

In this paper, the selected multi-dimensional feature parameters were used to estimate potato AGB in multiple periods by two regression algorithms (partial least squares regression, PLSR; random forest regression, RFR). The model precision was quantitatively characterized by R^2^, RMSE, MAE, and nRMSE (Dong et al., 2020) using a separate validation dataset (Figure 1C).

## Results

### Acquisition of SVs based on the SMA of dynamic endmembers

**Figure 2:**
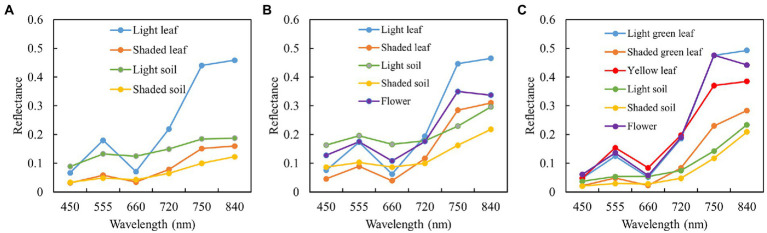
Field measured spectra of endmembers in different potato periods: **(A)** June 18; **(B)** July 17; **(C)** August 9.

The spectral endmembers in the field become more and more complex with the growth and development of potatoes. At SP, the light leaf (LL), shaded leaf (SL), light soil (LS), and shaded soil (SS) were included. At FP, the flower was added. At TP, the leaves can be divided into green and yellow ones. Thus, unlike FP, the light green leaf (LGL), shaded green leaf (SGL), and yellow leaf (YL) were added. By taking the mean value of measured spectral reflectance in the corresponding band range, spectral endmembers used for SMA in different periods are shown in Figure 2.

**Figure 3:**
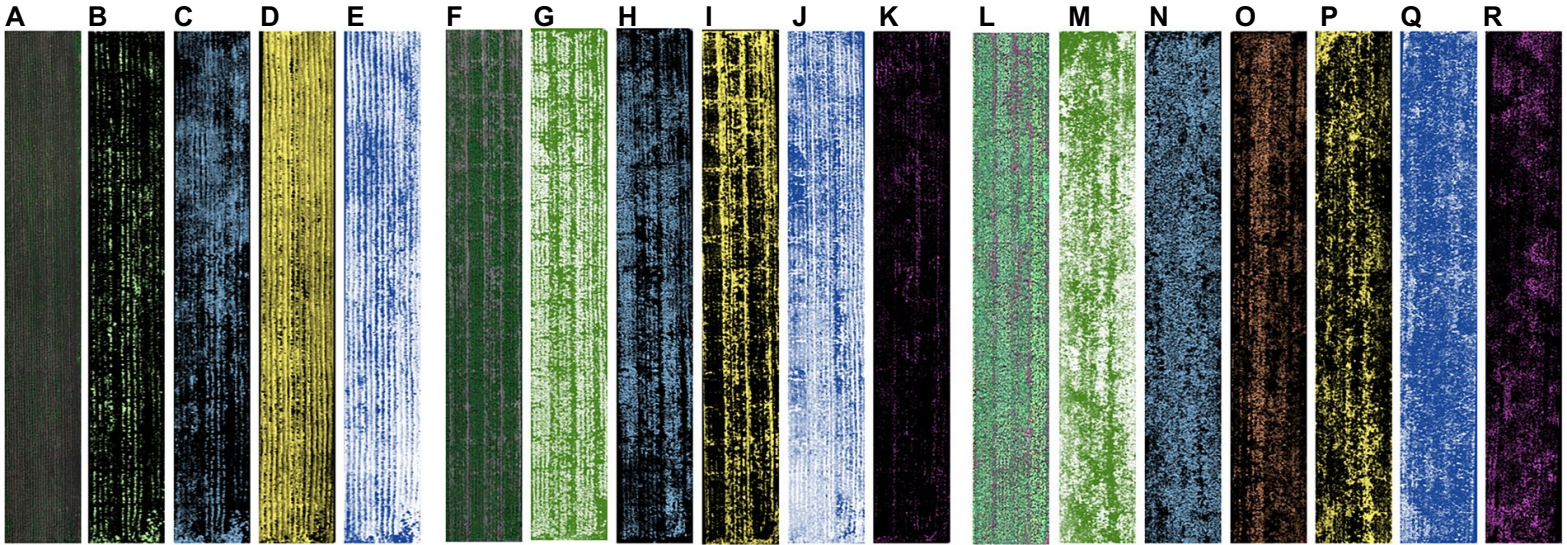
The abundance images of different potato growth stages: **(A–E)** LL, SL, LS, SS at SP; **(F–K)** LL, SL, LS, SS, flower at FP; **(L–R)** LGL, SGL, YL, LS, SS, flower at TP.

The results of LM-FCL-based SMA in different periods using dynamic endmembers are shown in Figure 3. It can be indicated that there are significant differences in abundance images of the same endmember at different stages (the more colored parts represents the greater abundance). For example, at SP, the colored parts of the leaf abundance (including LL and SL) are relatively lower than that at FP and TP and the soil abundance (including LS and SS) images show the opposite. Moreover, with the arrival of TP, the abundance of the flower and YL increases.

**Figure 4:**
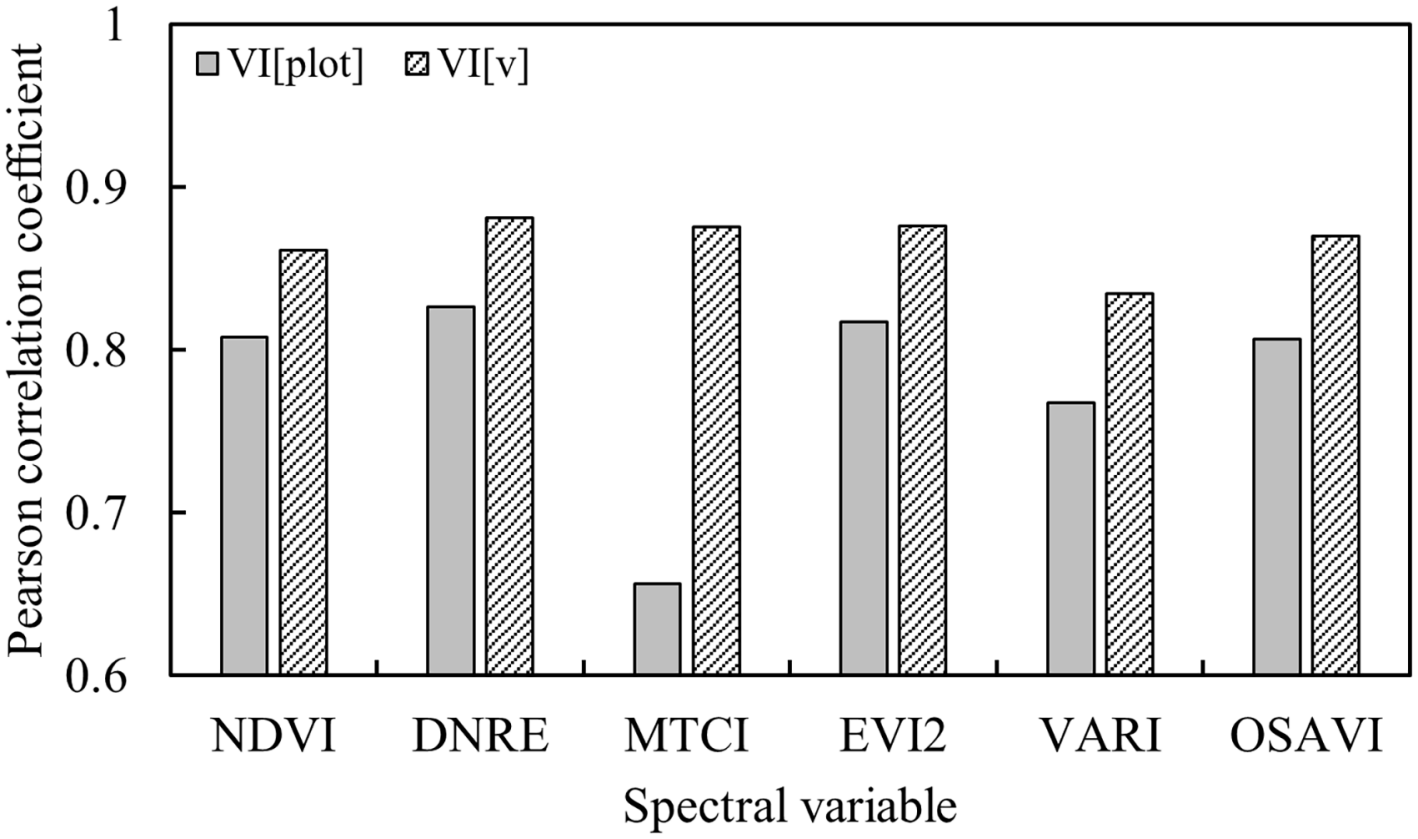
Correlation between potato AGB and VIs.

The VI calculated by the plot-level reflectance was defined as VI[plot]. To obtain the vegetation spectral parameters without soil background information, the product of the sum of abundances excluding soil and VI[plot] was defined as VI[v]. The correlation between potato AGB and VIs with different definitions is shown in Figure 4. It is seen that all listed VIs[v] were more correlated with the potato AGB than VIs[plot]. Thus, the VIs[v] were regarded as the SVs to predict the potato AGB.

### Extraction of TVs in different bands and computational directions

**Figure 5:**
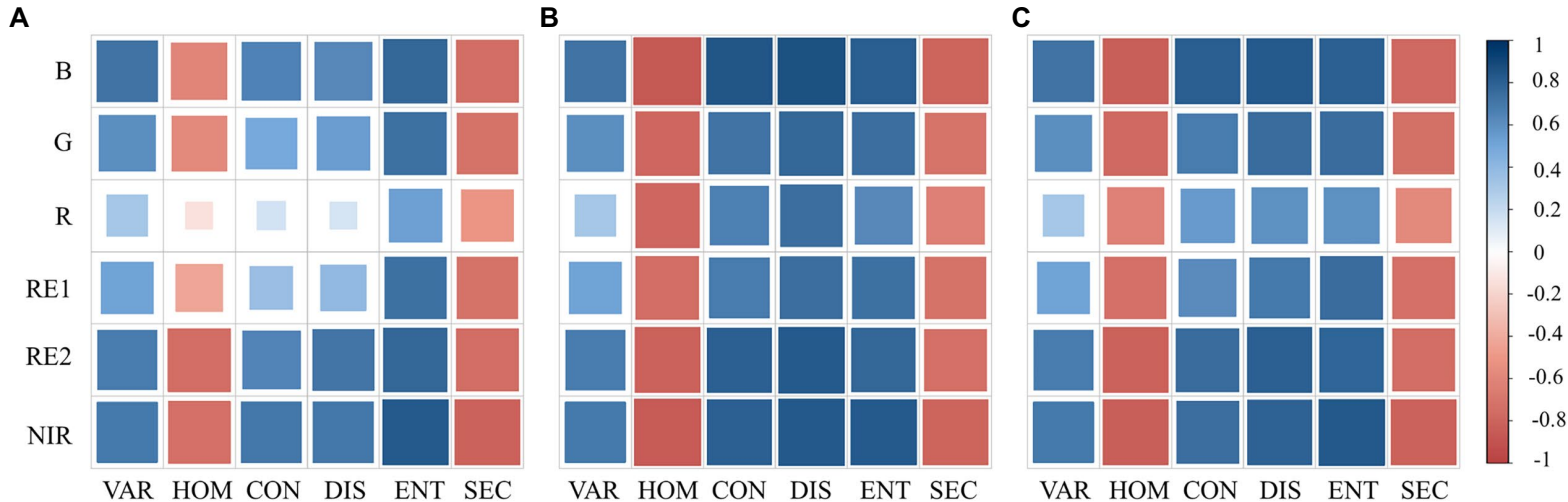
Correlation between potato AGB and textures based on different bands and calculation directions: **(A)** D_∥_; **(B)** D_⊥_; **(C)** D_∠_.

Six bands of the multispectral images were used to calculate textures in three different directions [parallel (D_//_) and perpendicular (D_⊥_) to the ridge, and an angle of 45° (D_∠_) to the ridge]. The correlation in Figure 5 shows that HOM and SEC were negatively correlated with AGB, while VAR, CON, DIS, and ENT were positively correlated with AGB. Furthermore, the correlation between VAR, ENT, SEC, and AGB was consistent in different directions, while the correlation between HOM, CON, DIS, and AGB was significantly different in three directions.

In terms of different bands, the B, RE2, and NIR-based textures had a high correlation with AGB. The textures of other bands showed instability in different directions. Therefore, the B-based textures in the D_⊥_direction with the highest correlation with AGB were referred to as TVs.

### Extraction and validation of GVs

**Figure 6:**
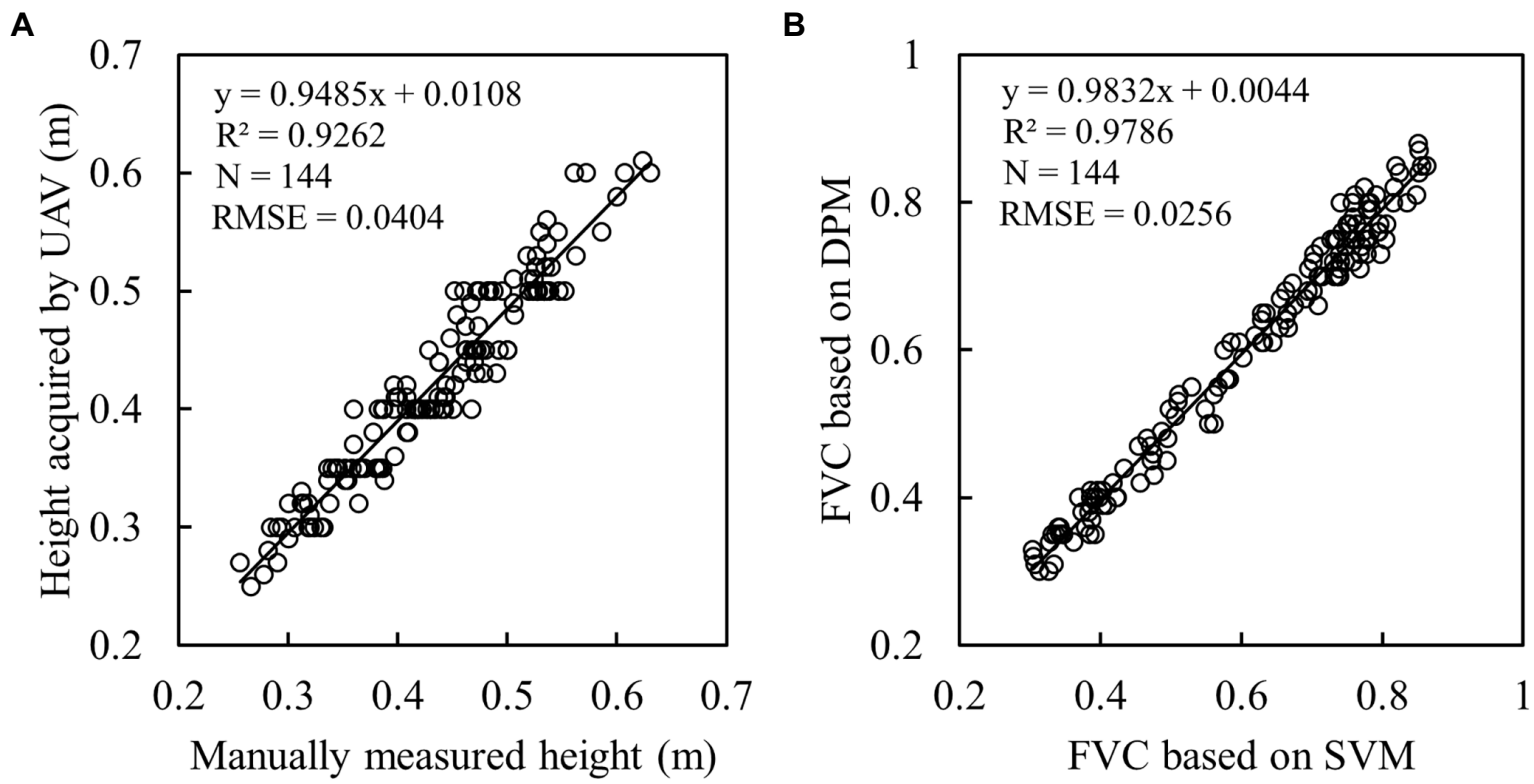
Verification of geometric parameters: **(A)** canopy height of potato; **(B)** canopy FVC of potato.

The UAV-based canopy heights were compared with the manually measured values (Figure 6A). The results showed that the heights acquired by UAV were highly correlated with the observed values (R^2^ = 0.9262, RMSE = 0.0404 m). In addition, the comparison of two methods of obtaining FVC (DPM and SVM) was performed (Figure 6B). It can be observed that the FVC obtained by these two methods has a good consistency (R^2^ = 0.9786 and RMSE = 0.0256). Therefore, we have reason to believe the accuracy of the FVC data extracted in this paper.

### Acquisition of FDVs based on harmonic decomposition

**Figure 7:**
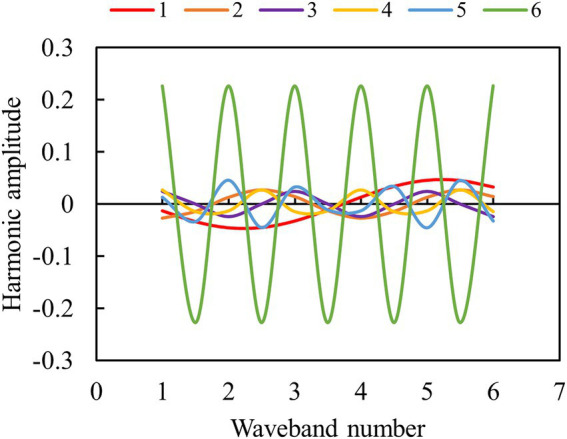
Schematic diagram of different harmonic decomposition times.

To explain the harmonic decomposition process, the spectra of an arbitrary potato plot were selected as an example, and the harmonic decomposition parameters were calculated six times according to the formula of C_t_sin(2πtj/n + φ_t_). Figure 7 shows that the maximum amplitude appears in the sixth decomposition, and the amplitudes of the first five decompositions show little difference. Different amplitudes can represent high- and low-frequency information in the spectra.

**Figure 8:**
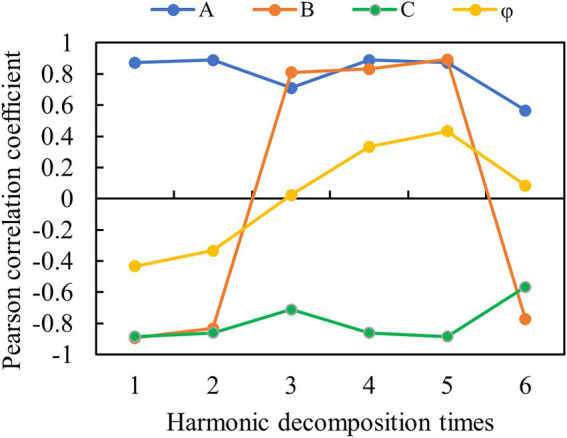
Correlation between potato AGB and harmonic parameters of different decomposition times.

The correlation between potato AGB and harmonic parameters of six decompositions is shown in Figure 8. The results indicated that except for the sixth decomposition, A and C obtained by the first five decompositions had a strong correlation with AGB. Also, the parameters of B obtained by six times of decomposition were strongly correlated with AGB. The correlation between φ and AGB obtained by all the decomposition times was weak. It concluded that low-frequency spectral information is more suitable for predicting potato AGB in multiple periods for FDVs.

### RRelieff and RF for feature variables selection

**Figure 9:**
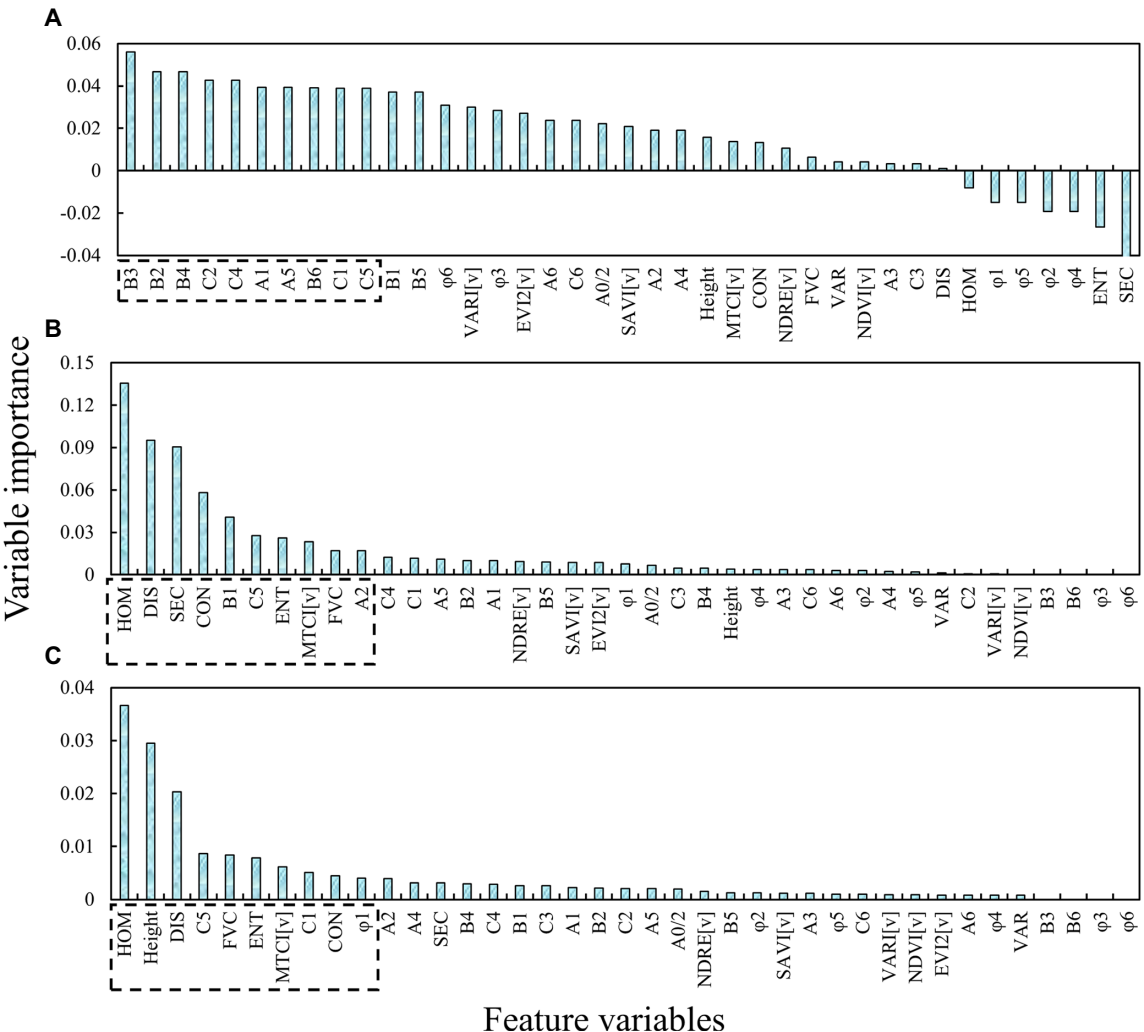
Variable importance ranking of different feature parameter selection methods: **(A)** RReliefF; **(B)** RF-Gini; **(C)** RF-OOB.

In this paper, 39 variables of spectra were extracted. And three feature selection algorithms were used to calculate the importance of different parameters. Figure 9 shows the ranking results of feature importance values. The top 10 feature variables were exhibited in the dotted box. The results indicate that there are great differences among the top 10 important indices extracted by the three methods, especially the RReliefF and RF-based methods. The importance of the variable calculated by RReliefF is based on the correlation with AGB. The higher the correlation, the greater the weight value. The top 10 important variables extracted by the two RF-based methods are very similar and parameters selected by RF-Gini are also highly correlated with AGB. However, the RF-OOB selected φ_1_ which is not highly correlated with AGB.

### AGB estimation using different regression algorithms and multi-dimensional variables

The AGB prediction accuracy of calibration and validation datasets are shown in Table 2. It can be found that the accuracy without any feature variable selection is similar to that based on RReliefF and RF-Gini for the calibration dataset, but there is an obvious difference in the accuracy of the validation dataset. The accuracy of all parameters-based models (including PLSR and RFR) is much lower than that of the RReliefF and RF-Gini-based models, which shows that amount of parameters for forecasting is not the more the better, and the redundant variables will reduce the robustness of the models.

**Table 2:** Potato AGB prediction results based on different feature selection methods and regression algorithms.

Feature selection methods	Regression methods	Calibration				Validation			
		*R* ^2^	RMSE (g/m^2^)	MAE (g/m^2^)	nRMSE (%)	*R* ^2^	RMSE (g/m^2^)	MAE (g/m^2^)	nRMSE (%)
None	PLSR	0.86	83.17	59.25	13.55	0.82	93.68	63.78	15.11
	RFR	0.87	80.34	58.51	13.08	0.83	90.79	63.61	14.64
RReliefF	PLSR	0.87	80.77	58.82	13.15	0.85	85.22	61.19	13.75
	RFR	0.88	77.21	55.82	12.57	0.87	80.48	58.59	12.98
RF-Gini	PLSR	0.85	85.32	61.25	13.90	0.84	88.30	62.23	14.24
	RF	0.87	80.45	58.66	13.10	0.85	85.64	61.50	13.81
RF-OOB	PLSR	0.89	73.54	53.85	11.98	0.88	77.15	56.37	12.44
	RF	0.91	68.76	49.18	11.20	0.90	71.68	51.27	11.56

In terms of the feature selection method, the variables extracted by RF-OOB have the highest prediction accuracy of potato AGB (R^2^ = 0.90, RMSE = 71.68 g/m^2^, MAE = 51.27 g/m^2^, and nRMSE = 11.56% for the validation dataset). From the perspective of the regression algorithm, the RFR has more advantages than PLSR for all variable selection scenarios in this paper.

**Figure 10:**
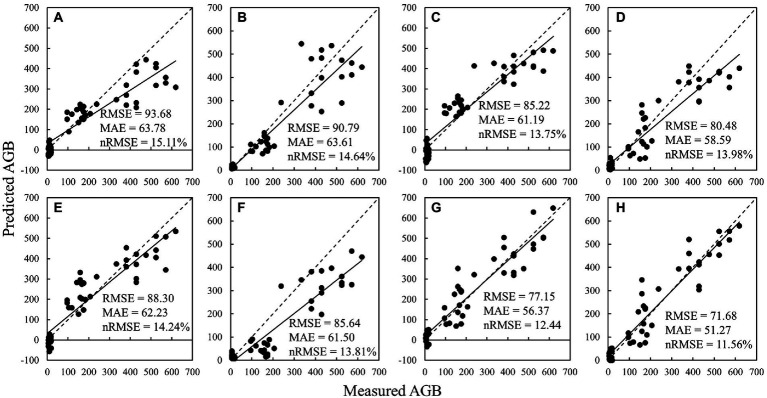
Comparison of measured and predicted AGB using different feature selection and regression algorithms: **(A)** None-PLSR; **(B)** None-RFR; **(C)** RReliefF-PLSR; **(D)** RReliefF-RFR; **(E)** RF-Gini-PLSR; **(F)** RF-Gini-RFR; **(G)** RF-OOB-PLSR; **(H)** RF-OOB-RFR.

A comparison chart of measured versus estimated AGB values in the validation dataset is shown in Figure 10 (the dashed line indicates the 1:1 line). It can be seen that for all feature selection methods (including no selection), the PLSR algorithm tends to produce negative values at low values of AGB. Moreover, the None, RReliefF, and RF-Gini-based models are prone to underestimate at high AGB values, especially in the range of 500–700 g/m^2^. The RF-OOB-based models are a good solution to the problem of underestimation of high-value AGB (the regression line almost coincides with the 1:1 line). Hence, the RF-OOB-RFR model works best for the estimation of multi-period potato AGB using multi-dimensional variables derived from multispectral imagery.

To measure the contribution of spectra and other variables to the potato AGB estimates, the variables of the four dimensions were combined into seven combinations. RF-OOB was then used to select the top ten most important parameters for modeling and validation (all variables would be selected if there were fewer than 10 variables). As shown in Table 3, for the same regression algorithm, the accuracy of different combinations increases with the increasing dimensionality of the variables. For the same combination of variables, RFR models have higher precision compared to that of PLSR, except for SV + TV. This suggests that the selections of variables and regression algorithms are equally important for AGB prediction.

**Table 3:** Potato AGB prediction results based on different variable combinations and regression algorithms.

Variable combinations	Regression methods	Calibration				Validation			
		*R* ^2^	RMSE (g/m^2^)	MAE (g/m^2^)	nRMSE (%)	*R* ^2^	RMSE (g/m^2^)	MAE (g/m^2^)	nRMSE (%)
SV + TV	PLSR	0.80	99.49	67.66	16.20	0.81	95.26	65.55	15.36
	RFR	0.82	93.74	63.82	15.27	0.80	98.93	67.04	15.96
SV + GV	PLSR	0.83	90.63	62.98	14.76	0.81	95.39	66.02	15.39
	RFR	0.85	85.29	61.30	13.89	0.83	90.56	63.28	14.61
SV + FDV	PLSR	0.82	93.87	64.02	15.29	0.81	96.33	66.89	15.54
	RFR	0.85	85.28	61.20	13.89	0.83	89.25	62.88	14.40
SV + TV + GV	PLSR	0.85	85.10	61.09	13.86	0.84	88.27	62.63	14.24
	RFR	0.87	80.54	58.75	13.12	0.87	80.86	58.97	13.04
SV + TV + FDV	PLSR	0.84	88.04	62.13	14.34	0.82	93.56	63.85	15.09
	RFR	0.88	77.39	56.04	12.60	0.86	83.26	59.34	13.43
SV + GV + FDV	PLSR	0.85	85.46	61.25	13.92	0.83	89.66	62.58	14.46
	RFR	0.88	77.03	56.15	12.55	0.87	80.61	58.31	13.00
SV + TV + GV + FDV	PLSR	0.89	73.54	53.85	11.98	0.88	77.15	56.37	12.44
	RFR	0.91	68.76	49.18	11.20	0.90	71.68	51.27	11.56

## Discussion

Crop AGB is an essential indicator of crop growth as well as crop breeding and management, and is one of the key factors affecting crop yield and profitability (Zhao et al., 2021). Potato has an irreplaceable role in ensuring food security, and the use of remote sensing technology to obtain potato AGB information can provide a basis for its yield estimation and provide decision-making information for farm production management and markets (Luo et al., 2020). The advent of remote sensing technology, especially UAV remote sensing, has made it possible to non-destructively and rapidly estimate crop AGB at the plot level (Osco et al., 2021).

Soil background or shadows can frequently affect the estimation of plant canopy parameters by radiation values (Wang et al., 2022). As shown in Figure 2, the spectral differences between components such as potato plants and background at different growth periods of potato were quite pronounced, and the proportion of different components (Figure 3) also changed significantly as can be seen by the abundance maps of each component. Therefore, spectral unmixing often results in good background removal when estimating crop parameters using spectral indices (Yang et al., 2007; Wang et al., 2022). The combination of VI and spectral unmixing results was often used to enhance the prediction of pure spectra (Zhou et al., 2018; Duan et al., 2019). In this paper, the product of VI and the abundance of vegetation was used to characterize the spectral information of potato, and the results showed that the correlation with AGB was significantly improved based on VI[plot] (Figure 4).

Moreover, the computational window scale has been shown no impact on the estimation of AGB when it comes to texture calculations (Li et al., 2020), but the choice involving specific orientation has been less reported. The results in Figure 5 demonstrated that among the six selected textures, VAR, ENT, and SEC are not affected by the computational direction, which is due to the fact that the computational equations of these three textures contain texture statistics reflecting the inside of the computational window, and a change in the computational direction does not cause a change in them, nor does it cause a change in the statistical values of all pixels within the window. In contrast, the calculation equations of HOM, CON, and DIS all contain information in different calculation directions, reflecting the texture statistics in the calculation direction within the window. Therefore, when there is an obvious texture pattern with direction in the image, a change in the calculation direction will have an obvious effect on these three textures. Potatoes are a planted-by-ridge crop, and their field morphology is directional, especially in the first and middle stages. Initially, there was a flat soil background in most of the canopy images, and seedlings only accounted for a small portion (Figure 3A), at which time the image homogeneity was high and heterogeneity was low. As the plants grow, seedlings grow a large number of new leaves in all directions, and the proportion of soil background decreases and the proportion of disordered leaves increases in the images (Figure 3B), leading to a weakening of image homogeneity and an increase in heterogeneity. After flowering, as well as the appearance of yellow leaves, the complexity was further increased (Figure 3C). Therefore, during the growth of rice, the homogeneity of images kept weakening and heterogeneity kept increasing with the accumulation of biomass, leading to a negative correlation between textures reflecting homogeneity (HOM and SEC) and biomass, and positive correlation between textures reflecting heterogeneity (VAR, CON, DIS, and ENT) and biomass. And the trend of this correlation does not change with orientations. The correlations of three directions suggested that the texture perpendicular to ridges reflected the potato growth information best (Figure 5). This may be due to the fact that the texture parallel to the ridges gives more expression to the spatial relationship between the potato plants, while the texture perpendicular to the ridges characterizes the relationship between the plants and the background, which is more indicative of the growth of the vegetation. It also provides a reference for texture selection of other ridge crops.

In addition to spectra and textures, parameters such as height and FVC are frequently exploited to improve the accuracy of crop biomass and yield estimation (Ashapure et al., 2020; Xu et al., 2022). In this study, the canopy height derived from DSM and DEM and FVC cross-validated by DPM and SVM (Figure 6) were obtained to participate in variable importance ranking and to improve the accuracy of AGB estimation. This is due to the fact that each of these parameters can characterize plant growth and development in different ways. For example, canopy height can provide stereoscopic information about the crop to compensate for the lack of canopy spectral information for estimating AGB (). LAI, which characterizes stereoscopic growth information of potato, was used to estimate yield and the results showed that its estimation was better than that of spectra (Luo et al., 2020). Therefore, more variables that can characterize plant stereo information (e.g., parameters obtained by LiDAR) are worth exploring for estimating crop AGB and yield. FVC, which represents the lushness of plant growth, has good parameter estimation ability, especially before crop closure of the canopy (Wan et al., 2020). Moreover, harmonic parameters were shown to be effective in crop biophysical parameter inversion (Zhuo et al., 2020; Jiang et al., 2021). However, the application of harmonic parameters in biomass estimation has been rarely reported. The results in Figures 7, 8 show that parameters highly correlated with the AGB could be extracted from both high-frequency and low-frequency spectral information. After the parameters of four dimensions were extracted, feature parameter selection becomes a new challenge (Faris et al., 2018).

Feature selection is critical in crop yield prediction, parameter inversion, and data preprocessing strategy, and overly redundant variables can even lead to reduced model robustness and accuracy (Li et al., 2018a, 2020). Thus, in potato biomass estimation, direct prediction of variables with multiple dimensions is evident to be inappropriate and necessary for feature selection. The results based on the three feature parameter selection methods show that RReliefF mainly conducts variable sorting according to the correlation with the target parameter (de Oliveira et al., 2017), which will lead to the failure to remove redundant features effectively and reduce the robustness of the model. RF-Gini, while similar to RF-OOB, leaves out important parameters such as height. Height has been shown to perform a vital part in AGB and yield estimation (Li et al., 2020; Maimaitijiang et al., 2020; Wan et al., 2020), which limits the accuracy of the model. The parameters selected by RF-OOB include not only highly correlated variables but also parameters such as height and φ_1_, although the correlation with AGB may not be high (Figure 9). This method mainly aims to reduce the error of the model.

The results of RF-OOB-RFR demonstrate that there is informational variability and complementarity between the parameters of different dimensions and that all these indices contribute to the estimation of AGB to different degrees. Additionally, RF-OOB algorithms are good at proposing indices with complementary information from parameters of different dimensions for the accurate estimation of AGB (Figure 10). This study can contribute to a scientific basis for timely and lossless monitoring of AGB in potatoes and other crops.

## Conclusion

In this study, four dimensions of variables (SV, TV, GV, and FDV, see Table 4) and three methods of feature parameter selection (RRreliefF, RF-Gini, and RF-OOB) were used to analyze and compare the estimation accuracy of potato AGB. When extracting parameters in different dimensions from the UAV images, the LM-FCL-based SMA method using dynamic endmembers was found to be effective in removing the influence of background, thus improving the correlation between VIs and AGB. In addition, the B-based textures in the D_⊥_ direction could show the ridge distribution of potatoes well. Variables of different dimensions were subsequently exploited for PLSR and RFR modeling and validation. It was found that the accuracy of the models continuously improved with the addition of variables of different dimensions, but this happened with the feature variable selection. Without any variable selection, the robustness of the model was very poor. Furthermore, the PLSR was prone to produce negative values at low values of AGB, while the RFR models could accurately predict AGB, especially when using four-dimensional variables and RF-OOB, and the underestimation problem for high values of AGB was well solved. According to the above results, the RFR model combined with four-dimensional variables and RF-OOB proposed in this paper is promising for accurate prediction of AGB and provides technical and theoretical support for rapid extraction of remote sensing phenotypic information of crops and high-throughput screening of plant phenotypes.

**Table 4:** The short glossary of terms in this study.

Full spelling words	Abbreviated glossary	Full spelling words	Abbreviated glossary
Aboveground biomass	AGB	Variance	VAR
Coefficient of determination	*R* ^2^	Homogeneity	HOM
Root mean square error	RMSE	Contrast	CON
Mean absolute error	MAE	Dissimilarity	DIS
Normalized RMSE	nRMSE	Entropy	ENT
Unmanned aerial vehicle	UAV	Second moment	SEC
Spectral variable	SV	Dimidiate pixel model	DPM
Vegetation indices	VIs	Out-of-bag	OOB
Texture variable	TV	Decision tree	DT
Gray level co-occurrence matrix	GLCM	Light leaf	LL
Geometric variable	GV	Shaded leaf	SL
Fractional vegetation cover	FVC	Light soil	LS
Frequency-domain variable	FDV	Shaded soil	SS
Random forest	RF	Light green leaf	LGL
Flowering period	FP	Shaded green leaf	SGL
Tuber period	TP	Yellow leaf	YL
Spectral mixture analysis	SMA	Partial least squares regression	PLSR
Linear model of fully constrained least-square	LM-FCL	Random forest regression	RFR

## Data availability statement

The raw data supporting the conclusions of this article will be made available by the authors, without undue reservation.

## Author contributions

SL wrote the manuscript. YH and JL provided the study ideas and completed the experimental design. WJ provided suggestions and edited the manuscript. SZ, FX, ZH, and JS measured the experimental data. JY and XW conducted the UAV flights. XM and ZL provided comments on the revision of the manuscript. All authors contributed to the article and approved the submitted version.

## Funding

This work was supported by the National Natural Science Foundation of China‘s “Study on temporally and spatially precise assessment on potato cultivation suitability based on dynamic process-oriented mode” (41771562) and “Innovation Project” of the Chinese Academy of Agricultural Sciences (2021–2025, IARRP).

## Conflict of interest

The authors declare that the research was conducted in the absence of any commercial or financial relationships that could be construed as a potential conflict of interest.

## Publisher’s note

All claims expressed in this article are solely those of the authors and do not necessarily represent those of their affiliated organizations, or those of the publisher, the editors and the reviewers. Any product that may be evaluated in this article, or claim that may be made by its manufacturer, is not guaranteed or endorsed by the publisher.
